# Ambient air pollution exposure and COVID-19 related hospitalizations in Santiago, Chile

**DOI:** 10.1038/s41598-024-64668-3

**Published:** 2024-06-20

**Authors:** Robert Dales, Anna O. Lukina, Rafael Romero-Meza, Claudia Blanco-Vidal, Sabit Cakmak

**Affiliations:** 1 Environmental Health Science and Research Bureau, Health Canada, 251 Sir Frederic Banting Driveway, Ottawa, ON, K1A 0K9 Canada; 2 University of Ottawa and Ottawa Hospital Research Institute, Ottawa, Canada; 3 School of Economics and Business, Universidad Alberto Hurtado, Santiago, Chile; 4 Department of Economics, Boston University, Boston, USA

**Keywords:** Chile, COVID-19, Hospitalization, Air pollution, Epidemiology, Epidemiology, Infectious diseases

## Abstract

Morbidity and mortality from several diseases are increased on days of higher ambient air pollution. We carried out a daily time-series analysis with distributive lags to study the influence of short-term air pollution exposure on COVID-19 related hospitalization in Santiago, Chile between March 16 and August 31, 2020. Analyses were adjusted for temporal trends, ambient temperature, and relative humidity, and stratified by age and sex. 26,579 COVID-19 hospitalizations were recorded of which 24,501 were laboratory confirmed. The cumulative percent change in hospitalizations (95% confidence intervals) for an interquartile range increase in air pollutants were: 1.1 (0.2, 2.0) for carbon monoxide (CO), 0.30 (0.0, 0.50) for nitrogen dioxide (NO_2_), and 2.7 (1.9, 3.0) for particulate matter of diameter ≤ 2.5 microns (PM_2.5_). Associations with ozone (O_3_), particulate matter of diameter ≤ 10 microns (PM_10_) and sulfur dioxide (SO_2_) were not significant. The observed effect of PM_2.5_ was significantly greater for females and for those individuals ≥ 65 years old. This study provides evidence that daily increases in air pollution, especially PM_2.5_, result in a higher observed risk of hospitalization from COVID-19. Females and the elderly may be disproportionately affected.

## Introduction

On April 21, 2024 WHO reported 775,364,261 confirmed COVID-19 cases, including 7,046,320 confirmed deaths globally^1^. In the United States alone, a total of 103,436,829 confirmed COVID-19 cases, as well as 1,186,079 deaths due to COVID-19 were recorded as of April 2024^2^. In Chile, since the start of the pandemic to December 19, 2023, there have been 5,339,561 confirmed cases of COVID-19 and 62,669 confirmed COVID-19 related deaths^1^.

Apart from host and virus-related characteristics that increase the risk of COVID-19 morbidity^3–7^, previous population-based studies suggest that ambient air pollution may be a risk factor for COVID-19 related hospitalization^8–10^. Most of these studies compared hospitalization rates with long-term exposure in different geographic regions^9,11–14^. Findings are susceptible to confounding by differences between the populations being compared, which may include: age, sex, burden of comorbidities, social deprivation^15^, population density^16^, climate^17^, and access to health care^17,18^. Confounding by differences between study groups can be largely avoided by using a time-series analysis design^15,19,20^, which can test the association between day-to-day changes in air pollution, and day-to-day changes in hospitalization or mortality rates in the same population. This model assumes that population characteristics do not change on a very short-term basis, and both measured and unmeasured risk factors remain stable between higher and lower air pollution concentration days. One possible confounding factor could be weather, which changes day-to-day and may be associated with overall air quality.

As part of a recent review of air pollution and COVID-19, Hernandez Carballo et al. (2022), evaluated 17 publications, based on 11 studies of hospitalization or non-fatal severity. Seven were classified as having positive results, eight had mixed results, and two were classified as having non-significant results^9^. The majority of studies reported health effects from exposure to ambient fine particulate matter with particle diameter of 2.5 µm or less (PM_2.5_) and nitrogen dioxide (NO_2_). One study assessed health effects from exposure to ambient ozone (O_3_) and sulfur dioxide (SO_2_) pollutants. Most studies were based on spatial differences in morbidity and only a minority of studies used a time-series design. A daily time-series study in 2020 reported that NO_2_ was positively associated with COVID-19 related hospitalizations in six of nine Spanish regions, and fine particulate matter with particle diameter of 10 µm or less (PM_10_) in four Spanish regions^21^. Another population-based study done in 2020, using simple correlation coefficients found that a 5-day moving average of daily mean PM_2.5_ levels lagged by 20 days was positively associated with critical care admissions in Florence and Milan, but not in Trento province^17^. Diaz et al. (2021) reported that hospitalizations in 2020 in Madrid were higher on days of higher NO_2_ concentrations, but not on days of higher PM_10_ concentrations^22^. We found only one time series analysis of air pollution and hospitalization south of the equator where winter, a time of enhanced respiratory viral transmission, occurs at a different time of the calendar year than north of the equator. In a municipality of Sao Paulo, Brazil, Santos et al. (2022) reported positive associations between hospitalizations and O_3_ lagged 2 days, and NO_2_ lagged 3 days^23^. No effect was found with PM_10_ and PM_2.5_ was not mentioned. The present study investigated the association between acute changes in the ambient air pollutant concentrations, PM_2.5_, NO_2_, O_3_, SO_2_, PM_10_ and carbon monoxide (CO), and COVID-19 related hospitalizations in Santiago, Chile between 16 March and 31 August 2020, during the early stage of the COVID-19 pandemic.

## Methods

### Study population

**Figure 1:**
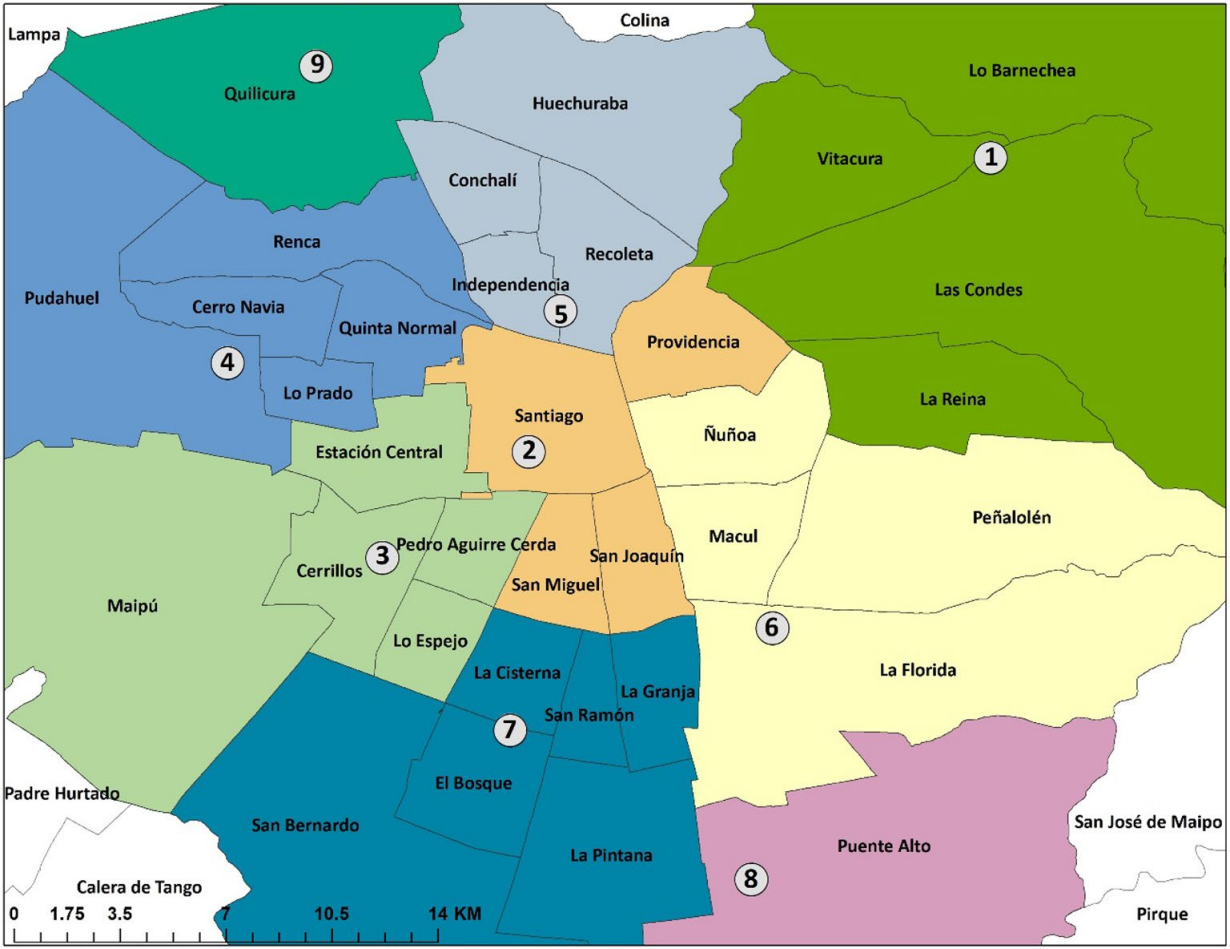
Map of Santiago, Chile. The numbers represent the location of air pollution monitoring stations in each of the nine colour-coded sectors: (1) Las Condes, (2) Parque O´Higgins, (3) Cerrillos, (4) Pudahuel, (5) Independencia, (6) La Florida, (7) El Bosque, (8) Puente Alto, (9) Quilicura. (Souce: Santiago Province in Chile, Census Data, 2020). The map was created using: ArcGIS Desktop 10.6 (https://www.esri.com/en-us/arcgis/products/arcgis-desktop/resources).

We included residents of the 32 comunas of the Province of Santiago, Chile and two adjacent urban comunas, Puente Alto, and San Bernardo. Figure 1, the regional map has been previously published^20^. The comunas were grouped into nine sectors, as following: Cerrillos, El Bosque, Independencia, La Florida, Las Condes, Parque O´Higgins, Pudahuel, Puente Alto, and Quilicura (Table 1).

**Table 1:** COVID-19 related daily hospitalizations and mean (IQR) of ambient 24-h mean air pollution concentrations, overall and by sector in a period between March 16 and August 31, 2020^20^.

Sector	Population	Mean Hospitalizations	CO	NO_2_	O_3_	PM_2.5_	PM_10_	SO_2_
		(daily)	(ppm)	(ppb)	(ppb)	(µg/m^3^)	(µg/m^3^)	(ppb)
1) Cerrillos	85,026	28.76	n/a	n/a	n/a	36.43 (22.0)	79.02 (45.5)	n/a
2) El Bosque	170,801	24.58	0.90 (0.56)	27.79 (12.26)	9.78 (10.0)	36.30 (22.0)	80.91 (37)	3.31 (0.86)
3) Independencia	105,437	17.15	0.79 (0.40)	6.94 (2.81)	9.74 (10.0)	28.70 (16.5)	68.30 (31)	n/a
4) La Florida	386,307	17.07	0.80 (0.38)	26.0 (11.39)	24.71 (6.0)	27.05 (15.0)	60.06 (26)	14.06 (8.99)
5) Las Condes	307,708	8.54	0.44 (0.19)	21.01 (11.04)	16.33 (10.5)	17.78 (12.0)	45.55 (27.5)	n/a
6) Pudahuel	240,958	21.33	0.90 (0.77)	16.44 (6.93)	10.96 (10.0)	35.66 (26.5)	78.33 (43)	n/a
7) Puente Alto^a^	568,094	15.69	0.61 (0.33)	14.48 (10.59)	13.73 (10.0)	27.09 (14.0)	67.53 (25)	n/a
8) Quilicura	222,048	3.92	n/a	n/a	n/a	30.80 (18.5)	66.35 (33)	n/a
9) Parque O´Higgins^b^	446,490	21.17	0.64 (0.41)	26.58 (12.83)	36.43 (22.0)	28.23 (19.0)	78.88 (36)	9.69 (2.07)
Overall	2,532,869	158.21	0.73(0.43)	19.89 (9.69)	14.21 (9.42)	29.78 (18.39)	69.44 (33.78)	9.02 (3.35)

n/a: pollution was not available at that location.

The population is based on 2017 Chile Population and Housing Census conducted in April 2017 (Santiago Province in Chile, Census data, 2020).

^a^Puente Alto is the capital of the Cordillera Province in the Santiago Metropolitan Region.

^b^Parque O´Higgins is a large park situated in the center of the capital city, within the Santiago Commune.

### Hospitalization data

Daily hospital discharge data recorded between March 16th and August 31st, 2020 were obtained from the Chilean Statistical Institution and provided by the Ministry of Environment of Chile. The *International Classification of Disease* 10th Revision (ICD-10)^24^ codes a laboratory confirmed diagnosis of COVID-19 as U07.1 and “a clinical or epidemiological diagnosis of COVID-19, where laboratory confirmation is inconclusive or not available” as U07.2. In the present study, either of the two codes was considered.

### Air quality data

Each of the nine sectors (Fig. 1) has a stationary ground-based air quality monitor from which we obtained 24-h average concentrations for PM_2.5_, NO_2_, O_3_, PM_10_, SO_2,_ and CO. For Cerrillos and Quilicura sectors, only PM_2.5_ measurements were available. SO_2_ was only available for three sectors. Twenty-four hour mean temperature and relative humidity were also collected from the ground-based monitors. Hospitalized cases were linked to ambient air pollution and weather variables by each sector of residence.

### Statistical analyses

The statistical analysis has been previously described^20^. Daily variations in the number of COVID-19 related hospitalizations were related to daily variations in ambient air pollutants concentrations by a two-stage random-effects regression model for count data as described in Cakmak et al. (2007), which assumes a Poisson distribution^19^. A linear association between air pollution and hospitalization on the logarithmic scale was assumed, with the association varying at random between sectors^19,25^. In the first stage, sector-specific regression parameter estimates of the pollution–hospitalization association were obtained. In the second stage, these estimated sector-specific regression parameters were then pooled using random-effects models^26^. First, separate analyses were used for each sector. Unwanted temporal trends (e.g., epidemics from viral respiratory illnesses) in daily hospitalization were addressed by sector using natural nonparametric splines functions for day-of -study and testing knots for every 15, 30, 45, and 60 days of observation. Model selection was based on minimization of the Akaike information criterion (AIC), a measure of model prediction. Additionally, Bartlett’s test was used to demonstrate that the model residuals did not display any type of structure, including serial correlation. Having selected the optimal model for time, we then tested 24-h mean temperature and relative humidity on the day of hospitalization and up to 6 days before hospitalization, for inclusion in the model using stepwise backwards and forwards regressions, while keeping the variables for time and day-of-the-week constant. Potential nonlinear associations of weather with hospitalization were addressed using natural spline functions. We examined linear weather models and models with up to 4 knots. The model that minimized the AIC was selected as the optimal weather model for each sector separately. An indicator variable in the models represented day-of-the-week to account for any possible differences in hospitalization rates between days of the week. Finally, we tested the effect of air pollution on the day of admission and the previous six days exposure using unconstrained distributed lags as described by Schwartz (2000)^27^. In the second stage, the regression parameter estimates assessing the associations between pollution and COVID-19 hospitalization within each sector were pooled using a random effects model^26^. Factors, such as age, sex, social status, and population density, were not adjusted for because they were assumed to be time invariant over the relatively short duration of the study. Sector specific estimates were pooled using the random effects model to calculate the overall effect sizes to air pollution related COVID-19 hospitalizations. In addition, two-pollutant models, each of which included adjustment for one of co-pollutants, were fitted. The association of air pollutants concentrations with hospitalizations was considered robust if the effect estimates in the single-pollutant and two-pollutant models were not significantly different, as determined by non-overlapping confidence intervals which indicates the differences were not significant at *p* < 0.05. To assess the robustness of the results to the lag structure used, we calculated lag 0 (or same day exposure) effects. Results, overall and individual, were stratified by age and biological sex. Results were presented as the pooled percent change in COVID-19 hospitalization for an interquartile range (IQR), the 25th to the 75th percentiles, concentration of each ambient pollutant. This nonparametric statistic naturally excludes extreme values, which could skew the estimate of effect. The data were analysed using an R program version 4.0.2^28^.

## Results

26,579 COVID-19 related hospitalizations occurred between March and August 2020 of which 24,501 were laboratory confirmed, resulting in a daily average of 158 cases. The median age of participants was 62 years old and 59% were males. Mean air pollution concentrations (IQR) were 0.73 ppm (0.43) for CO, 19.89 ppb (9.69) for NO_2_, 14.21 ppb (9.42) for O_3_, 29.78 µg/m^3^ (18.39) for PM_2.5_^20^, 69.44 µg/m (33.78) for PM_10_, and 9.02 ppb (3.35) for SO_2_ (Table 1). El Bosque had the highest recorded NO_2_ and PM_10_ levels, Cerrilos had the highest PM_2.5._ O_3_ was highest in Parque O’Higgins, and CO was highest in El Bosque and Pudahuel. The highest concentration of SO_2_, was recorded in La Florida (Table 1).

In the overall population of Santiago there were significantly increased risks (*p* < 0.05) of daily hospitalizations from COVID-19 for an IQR increase in CO, NO_2_, and PM_2.5_, of 1.1 (0.20, 2.0), 0.30 (0, 0.50), and 2.7 (1.9, 3.0), respectively, using distributive lags with cumulative percent changes in estimates (Table 2). The overall and sex-stratified effect sizes for O_3_ were not significant (*p* < 0.05) but subgroup analysis by age produced variable results with a significantly positive association for the youngest age group and a negative association among the 65–74 years old subgroup. The overall SO_2_ estimate of effect was not significant but there was a significant positive association for the > 84 years old age group and a significant negative association for the 65–74 year olds. Effect sizes for PM_2.5_, PM_10_, and SO_2_ were largest in the oldest age group. Compared to males, percent change in estimates for females were larger for CO, NO_2_, PM_2.5_, and PM_10_.

**Table 2:** Pooled cumulative percent change in (95%CI) of daily COVID-19 related hospitalizations for an IQR increase in ambient air pollutants using distributive lags over seven days in single pollutant models adjusted for ambient temperature and relative humidity.

		Ambient air pollutants						
		n (%)	CO	NO_2_	O_3_	PM_10_	PM_2.5_	SO_2_
Overall		26,579	1.1 (0.2, 2.0)*	0.3 (0.0, 0.5)*	− 1.1 (− 2.8, 0.0)	0.3 (− 0.4, 1.0)	2.7 (1.9, 3.0)*	− 2.4 (− 9.3, 4.0)
Sex	Male	15,683 (59.0)	0.6 (− 0.5, 1.7)	0.1 (− 0.2, 0.4)	− 0.6 (− 2.8, 1.7)	− 0.9 (− 1.8, 0.0)	1.5 (0.5, 2.6)*	− 4.5 (− 12, 3.6)
	Female	10,896 (41.0)	1.9 (0.5, 3.3)*	0.5 (0.1, 0.9)*	− 1.8 (− 4.4, 0.9)	2.1 (0.9, 3.2)*	4.4 (3.2, 5.7)*	− 0.6 (− 7.8, 7.1)
Age (years)	< 65	15,302 (57.6)	0.8 (− 0.4, 1.9)	0.2 (− 0.2 ,0.5)	2.6 (0.3, 5.0)*	− 0.3 (− 1.3 ,0.6)	1.9 (0.8, 2.9)*	− 1.8 (− 12.7, 10.4)
	65–74	5,884 (22.1)	1.5 (− 0.2, 3.3)	0.4 (− 0.1, 0.9)	− 9.9 (− 13.2, − 6.4)*	2.5 (1.1, 4.1)*	5.6 (4, 7.3)*	− 7.4 (− 10.7, − 4.0)*
	75–84	3,773 (14.2)	1.5 (− 0.9, 3.9)	0.5 (− 0.6, 1.7)	− 2.1 (− 6.6, 2.5)	2.6 (0.9, 4.3)*	5.6 (4.2, 7.1)*	− 1.6 (− 5.8, 2.8)
	> 84	1,620 (6.1)	1.6 (− 1.3, 4.5)	1.6 (− 1.3, 4.5)	− 1.8 (− 9.2, 6.3)	2.6 (1.2, 4.2)*	5.7 (4, 7.5)*	11.8 (4.4, 19.6)*

Results are presented for the overall population, and by biological sex and by age groups. Values marked with an asterisk are statistically significant (*p* < 0.05).

To test the sensitivity of the results to the lag structure used, we also calculated lag 0 (same day exposure) effects. Estimates were very similar to those using distributed lags with percent change in (95%CI) for CO, NO_2,_ and PM_2.5_ of 1.09 (0.20, 1.96), 0.30 (0, 0.50), and 2.63 (1.86, 3.38), respectively.

We tested the sensitivity of findings to the presence of co-pollutants using two pollutant models, CO lost its significance at *p* < 0.05 (confidence interval included 0) when either NO_2_ or PM_2.5_ were added to the model (Table 3). NO_2_ became non-significant when either PM_2.5_, PM_10_ or SO_2_ were added to the model. The SO_2_ effect became significantly negative when either CO, NO_2_, PM_2.5_, PM_10_ were added to the model.

**Table 3:** Pooled cumulative percent change in (95%CI) of COVID-19 hospitalization associated with an IQR increase in ambient air pollution concentrations in two-pollutant models using distributive lags over seven days. Models are adjusted for ambient temperature, relative humidity, and pollutants.

		Ambient air pollutants					
		CO	NO_2_	O_3_	PM_2.5_	PM_10_	SO_2_
Single pollutant model		1.1 (0.2, 2.0)*	0.3 (0.0, 0.5)*	− 1.1 (− 2.8, 0.0)	2.7 (1.9, 3.0)*	0.3 (− 0.4, 1.0)	− 2.4 (− 9.3, 4.0)
Pollutant added to model	CO	Na	2.1 (0.6, 3.7)*	− 0.2 (− 2.2, 1.8)	5.1 (2.9, 7.4)*	− 0.5 (− 1.4, 0.2)	− 2.0 (− 3.2, − 1)
	NO_2_	2.5 (− 0.5, 5.5)	Na	0.4 (− 2.8, 3.7)	3.7 (2.1, 5.3)*	− 0.2 (− 0.9, 0.4)	− 1.9 (− 3.0, − 0.9)
	O_3_	2.9 (0.2, 5.6)*	2.0 (0.5, 3.5)*	Na	1.6 (0.7, 2.5)*	0.0 (− 0.5, 0.5)	0.1 (− 0.7, 0.7)
	PM_2.5_	3.4 (− 1.6, 8.7)	− 0.7 (− 1.9, 0.7)	0.5 (− 3.0, 3.9)	Na	− 0.6 (− 2.8, 1.2)	− 1.9 (− 3, − 0.9)
	PM_10_	4.3 (0.0, 7.2)*	1.6 (− 2.0, 4.9)	− 0.5 (− 3.0, 2.2)	3.5 (1.7, 5)*	Na	− 1.9 (− 3, − 0.9)
	SO_2_	3.0 (0.8, 5.0)*	1.7 (− 0.3, 3.6)	− 0.5 (− 2.8, 2.0)	1.8 (1.1, 2.5)*	0.4 (− 0.1, 0.8)	Na

Values marked with an asterisk are statistically significant (*p* < 0.05).

Na-not applicable.

## Discussion

We found that, in single pollutant models, overall hospitalizations from COVID-19 increased on days of higher concentrations of ambient CO, NO_2_, and PM_2.5_. The effect of PM_2.5_ was robust, persisting despite subgroup analyses and adjustment for co-pollutants. Its observed effect was significantly less, *p* < 0.05, for males than females, and for those < 65 years old compared to those who were older. The observed PM_10_ effect was consistently non-significant across subgroups and in two-pollutant models. Estimates for O_3_ were not significant overall, in two -pollutant models, or when stratified by sex, and variable findings were noted in age subgroup analysis with significantly negative, significantly positive and non-significant results. The absence of SO_2_ measures for the majority of sectors together with stratification significantly reduced the sample size available for analysis. Unlike the robust effects of PM_2.5_, SO_2_ effects were inconsistent, with stratification increasing the probability of chance associations which were in different directions by age group.

Ambient air pollutants enhance oxidative stress and inflammation^29^ and have been associated with cardiac and pulmonary morbidity and mortality^30^, including an increased risk of respiratory infection^31^. The adverse effects of PM_2.5_ may lower the threshold for a COVID-19 infected person to deteriorate and require hospitalization^11^. In addition, PM_2.5_ activates the angiotensin converting enzyme (ACE) receptor to which SARS-CoV-2 attaches to gain access to cells^32^. Another hypothesis explaining why there may be an association between exposure to PM_2.5_ and COVID-19 related hospitalization is that particulate air pollution may be a vector for microbial transmission^33^, including possibly the coronavirus^34^. The short lag time observed in the present study would support the former hypothesis that air pollution may contribute to the burden of illness in someone already ill with COVID-19.

Previous population-based studies indicate that air pollution in Santiago, Chile, is associated with increased morbidity and mortality^19,20,35,36^, but the influence of air pollution on COVID-19 related hospitalization has not been previously studied. An ecologic study of continental Chile compared urban PM_2.5_ and PM_10_ concentrations estimated for the year 2016 to COVID-19 incidence and mortality in 2020 between communas normalized by the population size determined by a 2017 census. Adjusted incidence but not mortality was associated with the two air pollutants^37^. Outside of Chile, there have been several studies comparing hospitalization rates between geographic areas with different levels of ambient air pollution^9,10^. However, study designs comparing different populations are susceptible to confounding by regional differences in factors, such as age^38^, prevalence of underlying population comorbidities, socioeconomics^18,39^, urbanization/crowding^16^, weather^40,41^, and effectiveness of public health infection control measures. Focusing on Santiago, one study reported regional socioeconomic differences in the burden of illness due to COVID-19^42^. Rather than comparing morbidity between different geographically separated populations, our time-series analysis design contrasted the number of hospitalizations between higher and lower air pollution days in the same population in the same geographic area, thereby controlling for personal and geographic characteristics.

There are a few studies of short-term temporal association between air pollution and COVID-19 related hospitalization. A daily time series study done between February 1 and December 31, 2020, using lags of one, two, three, and four weeks found that NO_2_ was positively associated with daily COVID-19 related hospitalizations in six of eight Spanish regions, and PM_10_ and O_3_ in three regions^21^. Compared to Madrid, NO_2_ concentrations in Santiago were approximately double, 20.8 μg/m^3^ versus approximately 20 ppb (37.6 μg/m^3^), and the relative risks (RR) of hospitalization in our study was less than in Madrid 1.003 (95% CI 1.000, 1.005) per IQR (9.69 ppb) increase versus 1.007 (95% CI 1.006, 1.009) per 1 μg/m^3^ increase. Another population-based study conducted in the same year as our study, using simple correlation coefficients without comprehensively controlling for unwanted temporal variation, found that a five-day moving average of daily mean PM_2.5_ lagged by 20 days was positively associated with admissions to critical care in Florence, Italy (median PM_2.5_ 11.4 ug/m^3^) and Milan (median PM_2.5_ 17.8 μg/m^3^), but not in the Trento province^17^. NO_2_ was also positively associated with admissions in Trento province (median 18.3 ppb) and Florence (median 65 ppb) but not in Milan^17^. Diaz et al. (2021) reported that hospitalizations in 2020 in Madrid were higher on days of higher NO_2_ concentrations with a RR of 1.12 (95% CI 1.01, 1.24) per 10 μg/m^3^ increase, but not on days of higher PM_10_ concentrations^22^. If values were averaged over 14 days, hospitalizations were associated with PM_10_ with RR of 1.56 (95% CI 1.06, 2.20) per 10 μg/m^3^ increase, but not with NO_2_. The RR of hospitalization associated with NO_2_ were greater in Madrid than in Santiago highlighting the difficulty of trying to interpret associations between air pollution and COVID-19 hospitalizations based on comparisons between different geographic areas. Santos et al. (2022) using a time-series design, reported a relative risk of hospitalization to be 1.061 (95% CI 1.016–1.108) for O_3_ and 1.125 (95% CI 1.050–1.205) for NO_2_ in a municipality of Sao Paulo, Brazil^23^. There was no significant effect of PM_10_, and PM_2.5_ was not measured. We also found a positive effect of NO_2_ exposure and no significant effect of PM_10_. The largest effect size in our study was for PM_2.5_.

Strengths and Limitations: Our study has several relatively unique features. Previously, there have been very few time-series population-based studies of pollution-related COVID-19 hospitalization, and no studies were done in Santiago, Chile. Unlike other similar studies, we demonstrated the robustness of PM_2.5_ pollutant using two-pollutant models. To our knowledge, this is the first report of an association between COVID-19 related hospital admissions and the three ambient pollutants, CO, NO_2_, and PM_2.5_ using time series analysis to study short-term effects controlling for unwanted temporal variation. It is also one of the few such studies done in the southern hemisphere where the early pandemic occurred during the autumn rather than the early spring in the Northern hemispheres. The daily high and low temperatures in July are approximately 16 °C and 3 °C in Santiago, Chile. Winter weather with less sunlight, drier air, and lower temperatures may enhance virus survival^43^, and thus, modify the risk of COVID-19 related morbidity.

Being an observational study, the observed associations, in isolation, cannot be considered causal. However, the argument for causality is strengthened by previous publications of increased overall and COVID-19 related mortality on higher air pollution days in Santiago^19,20^, and the evidence for a plausible mechanism to explain the association^32^. The measurement error created by attempting to reflect personal exposure to air pollutants based on ambient stationary monitors is likely random and would tend to bias findings towards the null. This would suggest that the observed effect sizes are likely underestimated^44^. For our time series analysis we assumed that on average personal exposure is higher on days of higher ambient air pollution than on days of lower ambient air pollution and a previous study demonstrated positive correlations between ambient and personal exposure in Santiago^45^.

## Conclusion

Our study provides evidence of a significant association between short-term increases in ambient PM_2.5_, CO, and NO_2_ levels and daily COVID-19 related hospitalizations, with the most robust positive findings observed for PM_2.5_. These findings could help contribute to the overall body of evidence on air pollution and COVID-19 related illness and fill in knowledge gaps surrounding the short-term effects of ambient air pollution in an region south of the equator using a time series study design, which is relatively resistant to confounding by population-based characteristics. Furthermore, it could inform public health messaging and other efforts aimed at reducing air pollution, and possibly contribute to a reduction in the large burden of hospitalization from this novel coronavirus.

## Data Availability

The datasets used and/or analyzed during the current study are available from the corresponding author on reasonable request.

## Abbreviations

ACE: Angiotensin converting enzyme
AIC: Akaike information criterion
CI: Confidence intervals
CO: Carbon monoxide
IQR: Interquartile range
NO_2_: Nitrogen dioxide
O_3_: Ozone
PM_2.5_: Particulate matter of diameter ≤ 2.5 microns
PM_10_: Particulate matter of diameter ≤ 10 microns
RR: Relative risks
SO_2_: Sulfur dioxide
